# Is SF-12 a valid and reliable measurement of health-related quality of life among adults with Marfan syndrome? A confirmatory study

**DOI:** 10.1371/journal.pone.0252864

**Published:** 2021-06-09

**Authors:** Nathasha Udugampolage, Rosario Caruso, Mariangela Panetta, Edward Callus, Federica Dellafiore, Arianna Magon, Susan Marelli, Alessandro Pini

**Affiliations:** 1 Cardiovascular-Genetic Center, IRCCS Policlinico San Donato, San Donato Milanese, Italy; 2 Health Professions Research and Development Unit, IRCCS Policlinico San Donato, San Donato Milanese, Italy; 3 Clinical Psychology Service, IRCCS Policlinico San Donato, Milan, Italy; 4 Department of Biomedical Sciences for Health, Università degli Studi di Milano, Milan, Italy; Medical University Innsbruck, AUSTRIA

## Abstract

**Introduction:**

The structural validity and reliability of the Short-Form Health Survey 12 (SF-12) has not yet been tested in adults with the Marfan syndrome (MFS). This gap could undermine an evidence-grounded practice and research, especially considering that the need to assess health-related quality of life in patients with MFS has increased due to the improved life expectancy of these patients and the need to identify their determinants of quality of life. For this reason, this study aimed to confirm the dimensionality (structural validity) of the SF-12, its concurrent validity, and its reliability (internal consistency).

**Methods:**

We performed a cross-sectional study in a convenience sample of 111 Italian adults with MFS, collecting anamnestic and socio-demographic information, the SF-12, and short-form Health Survey 36 (SF-36). A confirmatory factor analysis was performed to verify whether the items of SF-12 related to physical restrictions, physical functioning, and bodily pain were retained by the physical summary component of the SF-12. The items referred to the role limitations due to emotional issues, social functioning, and mental health were retained by the mental summary component (MCS12). SF-36 was used to assess the concurrent validity of SF-12, hypothesizing positive correlations among the equivalent summary scores.

**Results:**

The two-factor structural solution resulted in fitting the sample statistics adequately. The internal consistency was adequate for the two factors. Furthermore, the physical and mental summary scores of the SF-36 were positively correlated with their equivalent summary scores derived from the SF-12.

**Conclusions:**

This study confirmed the factor structure of the SF-12. Therefore, the use of SF-12 in clinical practice and research for assessing the health-related quality of life among adults with MFS is evidence-grounded. Future research is recommended to determine whether the SF-12 shows measurement invariance in different national contexts and determine eventual demographic variation in the SF-12 scores among patients with MFS.

## Introduction

The Marfan syndrome (MFS) is an autosomal dominant systemic disorder of connective tissue caused by an altered mutation in the Fibrillin-1 gene (FBN1) [1]. The MFS has a prevalence ranging between 4.6 and 30 per 10.000 inhabitants [1]. Its clinical phenotypes encompass disorders, including the cardiovascular, skeletal, ocular, nervous, respiratory, and integument systems [2]. The most detrimental implications of MFS are related to the cardiovascular system, as MFS can lead towards an increased dilatation of the aorta, also increasing the risks for dissection of the thoracic vessels [2]. In patients with MFS, other physical features include hypermobile joints, thoracic deformities with scoliosis, arachnodactyly, general tapered, and slouching habitus [1].

Furthermore, the life expectancy in the population with MFS has been significantly improved over the last three decades [3], acknowledging the medical advancements in managing and preventing complications, especially for the cardiovascular system [3]. Recently, the literature has paid increased attention to the description of the health-related quality of life (HRQoL) in the population with MFS, even if its epidemiological descriptions are still lacking in many specific national contexts [4]. Given that the MFS is a potentially disabling disease, describing HRQoL and its determinants are pivotal for planning accurate educational and supportive actions [5].

The described socio-demographic determinants of HRQoL are mostly related to age and sex. Accordingly, inadequate HRQoL is more common in elderly patients and among males [5]. However, these studies are mainly referred to as specific geographic contexts, such as the United States of America (USA) and China; for this reason, caution is needed in generalizing the described socio-demographic determinants of HRQoL [5, 6]. Thus far, research on HRQoL was mainly performed by using the short-form Health Survey 36 (SF-36), as SF-36 showed evidence of validity and reliability in patients with MFS [4, 7]. Although a briefer version of SF-36 is available, i.e., Short-Form Health Survey 12 (SF-12), showing good psychometric proprieties in different cohorts of patients (e.g., general and disease-specific populations), the use of SF-12 in patients with MFS is not currently supported by evidence of validity and reliability. More precisely, SF-12 was sporadically used to assess HRQoL in the population with MFS, but the results of this research are limited by the need to provide proofs of validity in this specific population [4]. The use of the SF-12 is pivotal, considering that SF-36 could be time demanding in many clinical settings or studies employing multi-questionnaires. Previous research highlighted the necessity to perform confirmatory studies regarding the construct validity of the SF-12 when it has to be adopted in specific groups, as construct validity could be influenced by population-specific peculiarities [8, 9]. Validation studies could provide a solid basis for interpreting the evidence from empirical research performed using SF-12 in patients with MFS. Therefore, we aimed to confirm the dimensionality of the SF-12, its concurrent validity, and its reliability (internal consistency).

## Methods

### Design

This research is a cross-sectional study with a convenience sample of adults with MFS.

### Sample and procedure

All adult patients with the characteristics consistent with the inclusion/exclusion criteria, who were being treated in the specialized outpatient clinic for MFS at the IRCCS Policlinico San Donato Center, Milan (Italy), during 2019 and 2020 (the first four months), were asked to participate in the study (n = 142 adults with MFS). The inclusion criteria were: diagnosis of MFS [2], age equal to or higher than 18 years of age, ability to speak, read, and write in Italian. The presence of cognitive impairments was evaluated through the patients’ available clinical records, and they were considered as exclusion criteria. The outpatient clinic for MFS is a multidisciplinary service in which patients with MFS are seen by cardiologists, clinical geneticists, nurse practitioners, and psychologists throughout annual (or scheduled) follow-ups. This study reported a response rate of 78.2% (111 adults with MFS agreed to be enrolled). The sample size was considered as adequate for factor analysis, as SF-12 has a two-factor structure theoretically [10], and the “quality criteria for measurement properties of health status questionnaires” indicated that a sample size of roughly 50 participants per factor (domain) would be adequate for determining reliability and construct validity [11].

All eligible patients (n = 142) were contacted through e-mails containing the invitation to the study and an informative page with the study aim, procedure, and contact details in case of doubts or needs for further information. Patients who agreed to participate (n = 111) were invited to use a hospital web-based application, compliant with the European Union General Data Protection Regulation (GDPR), to electronically sign the informed consent and answer the self-report questions regarding the study. Self-report clinical or anamnestic data could be corroborated by the available information derived from the electronic medical records. The study procedure was consistent with previous research on HRQoL in different cohorts [12]. The Ethical Committee of Ospedale San Raffaele approved this study (project INDACO, prot. n. 62/int/2020).

### Measurements

The following anamnestic and socio-demographic characteristics were collected: age (years), sex (male, female), education (lower than high school diploma, equal to high school diploma, higher than high school diploma), and time from diagnosis (years). Furthermore, participants were asked to compile the SF-12 and SF-36. As per previous research [10], patients were initially asked to compile the SF-12, followed by the SF-36.

SF-36 is widely used to assess HRQoL using a self-report approach [13]. In this study, we used the Italian version of SF-36 [14]. SF-36 encompasses 36 items to assess physical functioning, role limitations due to physical problems, bodily pain, general health perceptions, vitality, social functioning, role limitations due to emotional problems, and perceived mental health [13, 14]. Each item has response categories which vary from two- to six-point scales; accordingly, raw scores for items range from 1 to 6, which require to be linearly transformed into two 0–100 components, named as physical component summary (PCS36) and the mental component summary (MCS36) [13]: a higher scores indicating a better health perception. Several studies reported that SF-36 has adequate internal consistency (the extent to which items within the scale’s components are correlated with each other) and adequate construct validity (SF-36 scores varied in relation to predefined hypotheses, such as it can discriminate different levels of HRQoL in patients who theoretically have different levels of HRQoL) [12].

SF-12 was developed to shorten the SF-36 using a regression method [10]. In this study, we used the Italian version of SF-12 [15]. As per the dimensionality of the SF-36, SF-12 encompasses 12 items that have to be scored in two 0–100 components: the physical component summary (PCS12) and the mental component summary (MCS12). Although the dimensionality of the SF-12 was never tested in patients with MFS, the literature has shown that it has adequate construct validity and reliability [8–10, 16]. SF-12 items are referred to as physical restrictions, physical functioning, bodily pain, general health compared with others, vitality, social functioning, role limitations due to emotional issues, and mental health (MH). The raw scores obtained from each item response have to be linearly transformed in a 0–100 scale to score PCS-12 and MCS-12 [10].

### Statistical analysis

A preliminary frequency check was performed to evaluate possible errors, outliers, and missing data. Descriptive statistics [mean, standard deviations (SD), percentage] were employed to describe the socio-demographic characteristics of the responders and the questionnaires’ items. The analysis of skewness and kurtosis was used for each discrete measure as a proxy evaluation to assess distribution. Missing data regarding the responses to the items ranged between 4.5% (5 participants skipped item 1) and 7.2% (8 participants skipped item 12). Considering that missingness was limited (the highest missing information was 7.2% referred to item 12), the full information maximum likelihood (FIML) estimation was employed to manage missing data, as considered appropriate as per the observed missingness [17]. As preliminary analyses, the evaluation of the associations between socio-demographic characteristics and PCS12/MCS12 have been conducted employing multivariable regression models expressing standardized estimates (β) and their relative 95% confidence intervals (95% CI) (see S1 Table).

As the SF-12 encompassed both categorical and discrete items, we adopted a robust estimator for determining the unknown parameter of the confirmatory factor analysis (CFA) within the framework of structural equation modeling (SEM), which was the Maximum Likelihood and Weighted Least Square Mean and Variance Adjusted (WLSMV) estimator [18] of Mplus. CFA allows researchers to test the theoretical structure of a scale, and it determines how well the scale model fits the empirical data (structure validity or dimensionality). Following previous studies [10, 19, 20], the items of SF-12 related to physical restrictions, physical functioning, and bodily pain were theorized to be retained by PCS12, whereas role limitations due to emotional issues, social functioning, and mental health were theorized to retained by MCS12. Therefore, following the approach of previous studies in patients with diverse health status and illnesses [8, 14, 16, 19, 20], we performed a CFA on the SF-12 responses to confirm the two-factor structure without constraining the effects of residuals, factor loadings, and factors were allowed to correlate. Factor loadings were reported using a completely standardized solution, and when >|.30|, they were considered adequate. We considered adding specifications to the model, evaluating possible estimates using the modification index (Lagrange multiplier) to assess whether adding any fixed parameters to the model would significantly reduce the model’s fit to the data [21]. The modification index is an estimate of the amount by which the χ^2^ would be reduced, improving the model’s fit, acknowledging that smaller χ^2^ values reflect that the model is adequate to explain the observed sample statistics (as well as the fit indices derived from χ^2^ statistics) when a single parameter restriction is removed from the model. However, modification index is a data-driven approach; for this reason, after a careful evaluation of the estimates, we preferred to consider the theory-driven approach [8, 14, 16, 19, 20], without applying specifications to correlate residuals (unconstrained model), i.e., the difference between the value of the observed sample statistic and the model-estimated value. The following goodness-of-fit indices were considered to evaluate the CFA: χ^2^ statistics [χ^2^ and χ^2^/degrees of freedom (df)], comparative fit index (CFI), Tucker and Lewis index (TLI), root mean square error of approximation (RMSEA), and standardized root mean square residual (SRMR). Overall, the criteria for considering adequate goodness of fit were: CFI and TLI values of.90–.95 indicate good fit; RMSEA values ≤.08 indicate adequate fit; values of SRMR ≤.08 indicate good fit as well [18]. Furthermore, we evaluated the possibility of a single-factor structure following previous research [19]. The difference of the model fit of the one-factor solution over the two-factor model was performed using nested model comparisons, performing the χ^2^ difference test by evaluating the χ^2^ difference (TRd), the difference between degrees of freedom (Δdf), and P-value for TRd. A significant difference in the χ^2^ statistic between the two models was suggestive of discriminant validity, as the model with the more satisfactory fit indices was more likely to be adequate in explaining the data.

Once identified the more satisfactory factor solution in explaining the sample statistics, the internal consistency of PCS12 and MCS12 was assessed through Cronbach’s α as a measure of reliability. After confirming the dimensionality of SF-12, we scored PCS12, MCS12, PCS36, MCS36. Pearson’s correlations between PCS12–PCS36 and MCS12–PCS36 were employed to determine the concurrent validity of SF-12, where positive correlations provide evidence of construct validity: Pearson’s correlation coefficients (r) were interpreted as being strong for r equal to or higher than 0.6, moderate for r ranging between 0.3 and 0.6, and low for r inferior to 0.3. The significance level of 0.05 was set for each inferential test. All statistical analyses were performed by using IBM Statistical Package for the Social Science (SPSS Inc., Chicago, IL, USA) version 22 and Mplus version 8.1 (Muthen & Muthen, 1998–2017).

## Results

The final study sample consisted of 111 adults with MFS (Table 1). Most of the participants were females (n = 66; 59.5%), and holding a high school diploma (n = 48; 43.2%). The mean (SD) age of the study sample was equal to 42.91 (12.19) years, and the mean (SD) time from MFS diagnosis was equal to 16.77 (14.09).

**Table 1:** Socio demographic characteristics of the sample (N = 111) and description of the domains of SF12 and SF36.

		*N*	%
Age			
	years (mean; SD)	42.91	12.19
Sex			
	Male	45	40.5
	Female	66	59.5
Education			
	Lower than high school diploma	28	25.2
	High school diploma	48	43.2
	Higher than high school diploma	35	31.5
Year from diagnosis			
	years (mean; SD)	16.77	14.09
	Yes	9	8.1
	No	80	72.1
	Don’t know	22	19.8
PCS36			
	Score (mean; SD)	65.83	36.73
MCS36			
	Score (mean; SD)	59.97	27.15
PCS12			
	Score (mean; SD)	45.93	10.79
MCS12			
	Score (mean; SD)	45.41	10.17

*Note*: SD = standard deviation.

The mean scores of PCS36 and MCS36 were 65.83(36.73) and 59.97(27.15), respectively. The skewness and kurtosis coefficients for all items (SF-12 and SF-36) were found to be within a range of– 2.511 and +0.973. The associations between socio-demographic characteristics and PCS12/MCS12 are described in S1 Table, showing that only age was associated with PCS12: the higher age, the lower PCS12 (β = -.324; 95%CI = -.464 –-.109; p = 0.002).

The first model assessed the two-factor solution, and it showed a satisfactory fit to the sample statistics: χ^2^_(53)_ = 101.83, p < 0.001; χ^2^/df = 1.9; RMSEA = 0.072, 90% CI (0.054–0.089); CFI = 0.944; TLI = 0.931; SRMR = 0.032. As described in Table 2, the standardized factor loadings of the items kept by PCS12 ranged between |0.662| and |0.742|; likely, the standardized factor loadings of the items kept by MCS12 ranged between |0.468| and |0.751|. The correlation between MCS12 and PCS12 was equal to 0.641 (P<0.001). After evaluating the possibility to constrain the residual correlations, the posed unconstrained model resulted as satisfactory.

**Table 2:** Psychometric evaluation of SF12: Confirmatory factor analysis (estimator = WLSMV).

	Mean ± SD	Frequency (per cent) of the ratings	CFA Factor Loadings	
			PCS12	MCS12
Item 1	3.24 0.94	Rating 1 = 2 (1.8%)	**0.742**	
		Rating 2 = 19 (17.1%)		
		Rating 3 = 49 (44.1%)		
		Rating 4 = 24 (21.6%)		
		Rating 5 = 12 (10.8%)		
		Missing = 5 (4.5%)		
Item 2	2.58 0.64	Rating 1 = 9 (8.1%)	**- 0.767**	
		Rating 2 = 27 (24.3%)		
		Rating 3 = 69 (62.2%)		
		Missing = 6 (5.4%)		
Item 3	2.41 0.63	Rating 1 = 8 (7.2%)	**- 0.662**	
		Rating 2 = 46 (41.4%)		
		Rating 3 = 50 (45.0%)		
		Missing = 7 (6.3%)		
Item 4	1.64 0.48	Rating 1 = 38 (34.2%)	**- 0.772**	
		Rating 2 = 67 (60.4%)		
		Missing = 6 (5.4%)		
Item 5	1.62 0.48	Rating 1 = 39 (35.1%)	**- 0.675**	
		Rating 2 = 64 (57.7%)		
		Missing = 8 (7.2%)		
Item 6	1.62 0.48	Rating 1 = 39 (35.1%)		**- 0.610**
		Rating 2 = 64 (57.7%)		
		Missing = 8 (7.2%)		
Item 7	1.56 0.49	Rating 1 = 46 (41.4%)		**- 0.602**
		Rating 2 = 58 (52.3%)		
		Missing = 7 (63.%)		
Item 8	2.25 1.09	Rating 1 = 33 (29.7%)	**0.795**	
		Rating 2 = 28 (25.2%)		
		Rating 3 = 29 (26.1%)		
		Rating 4 = 12 (10.8%)		
		Rating 5 = 2 (1.8%)		
		Missing = 7 (6.3%)		
Item 9	3.39 1.06	Rating 1 = 4 (3.6%)		**0.458**
		Rating 2 = 22 (19.8%)		
		Rating 3 = 20 (18.0%)		
		Rating 4 = 46 (41.4%)		
		Rating 5 = 12 (10.8)		
		Missing = 7 (6.3%)		
Item 10	3.84 1.06	Rating 1 = 13 (11.7%)		**0.751**
		Rating 2 = 23 (20.7%)		
		Rating 3 = 41 (36.9%)		
		Rating 4 = 22 (19.8%)		
		Rating 5 = 5 (4.5%)		
		Missing = 7 (6.3%)		
Item 11	4.26 0.97	Rating 1 = 1 (0.9%)		**- 0.468**
		Rating 2 = 4 (3.6%)		
		Rating 3 = 12 (10.8%)		
		Rating 4 = 47 (42.3%)		
		Rating 5 = 32 (28.8%)		
		Rating 6 = 9 (8.1%)		
		Missing = 6 (5.4%)		
Item 12	4.09 1.12	Rating 1 = 4 (3.6%)		**- 0.587**
		Rating 2 = 8 (7.2%)		
		Rating 3 = 13 (11.7%)		
		Rating 4 = 33 (29.7%)		
		Rating 5 = 45 (40.5%)		
		Missing = 8 (7.2%)		

**Model fit**: χ2(53) = 101.83, p < 0.001; χ2/df = 1.9; RMSEA = 0.072, 90% CI (0.054–0.089); CFI = 0.944; TLI = 0.931; SRMR = 0.032.

WLSMV = Maximum Likelihood and Weighted Least Square Mean and Variance Adjusted Estimators.

The second model tested the one-factor solution: χ^2^_(54)_ = 207.092, p < 0.001; χ^2^/df = 3.8; RMSEA = 0.164, 90% CI (0.140–0.187); CFI = 0.739; TLI = 0.682; SRMR = 0.102. The standardized factor loadings of items kept by the single factor were unsatisfactory, ranging between |0.182| and |0.860|. The χ^2^ difference test between the two-factor solution and the one-factor model was significant (TRd = -105.262; Δdf = 1; P<0.001), supporting the hypothesis of adequate discriminant validity of the two-factor solution, acknowledging its smaller χ^2^ value, more satisfactory fit indices, and a more suitable range of the standardized factor loadings. Therefore, the two-factor solution supported the scoring procedure considering that the items related to physical restrictions, physical functioning, and bodily pain were retained by PCS12, whereas MCS12 adequately retained the items related to role limitations due to emotional issues, social functioning, and mental health. The internal consistency of PCS12 and MCS12 were adequate, exhibiting the following Cronbach’s α, respectively: 0.892 and 0.897.

The mean (SD) scores of PCS12 and MCS12 were 45.93 (10.79), 45.41 (SD = 10.17). The correlation between PCS12 and PCS36 was highly positive and significant (r = 0.618; P<0.001); likely, the correlation between MCS12 and MCS36 was moderate and positive (r = 0.329; P<0.001). These correlations support the *a priori* hypotheses of positive relationships between PCS12–PCS36 and MCS12–PCS36, supporting the concurrent validity of SF-12 with the SF-36.

## Discussion

To the best of our knowledge, this was the first study aimed at confirming the dimensionality (structural validity), concurrent validity, and reliability of the SF-12 in adults with MFS. The evidence of validity and reliability of the SF-12 among patients with MFS is pivotal for both research and practice, also considering that the current healthcare scenario is characterized by increased attention towards the HRQoL of patients with MFS [4, 5, 7, 22, 23]. More precisely, the study of the determinants of HRQoL in patients with MFS is highly strategic for addressing supportive strategies in practice, as the impact of MFS on the individual could considerably differ between patients with similar clinical characteristics [5, 7]. In other words, some socio-demographic and psychological characteristics could influence directly or indirectly the HRQoL [5, 7].

This study confirmed that the original conceptual SF-12 model was adequate to explain the item-level sample statistics due to PCS12 retained items regarding physical restrictions, physical functioning, and bodily pain, and role limitations due to emotional issues, social functioning, and mental health were retained by MCS12 [8, 14, 16, 19, 20]. This evidence is important, as it also sustains the scoring procedure of SF-12 when it is used among patients with MFS. However, the debate on the scoring procedure of the SF-12 is still alive among researchers [24]. Previous studies showed that some model specifications were needed to improve the model’s fit-to-the-data: for instance, items 1, 10, and 12 were allowed to cross-load in both PCS12 and MCS12 [24], whereas some authors added residual covariances between items 2 and 3 (physical functioning), items 4 and 5 (role physical), items 6 and 7 (role emotional), and items 9 and 11 (mental health) [25, 26]. In the current study, we noticed that the fit of the model was satisfactory, without applying any model specification, and the two model factors showed adequate internal consistency; these results contribute to corroborate the theoretical structure of the SF-12.

We determined the model fit to data by analyzing in particular χ^2^ statistics, RMSEA, CFI, TLI, and SRMR. Given that χ^2^ statistic is highly sensitive to sample size [27], the evaluations of RMSEA, RMSEA, CFI, TLI, and SRMR are important to determine whether the hypothesized model fit the data well. All these indices are based on a fit-function that is specific to the chosen estimation method that, in this study, was the WLSMV. Thus far, the validation studies of SF-12 used diverse estimation methods for the factor analyses [8, 14, 16, 19, 20]. In the current study, the choice to adopt the WLSMV estimation method for determining the unknown parameter of the model was based on its described adequacy in evaluating the factor structure of categorical or ordered-categorical items, as per the SF-12 [28]. A previous simulation study demonstrated that the WLSMV estimation could lead to smaller RMSEA and larger CFI and TLI values [18]. However, in this study, the two-factor solution showed adequate indexes related to RMSEA, CFI, TLI, supporting the two-factor structural validity of the SF-12 in adults with MFS.

The scoring procedure was performed following the original scoring algorithm of SF-12 [10]. Nonetheless, we have to acknowledge that some concerns have been raised on the SF-12 scoring procedure, as some authors proposed to adopt population-specific and SEM-based scoring algorithms for scoring the summary components [26]. The main concern remains the method of deriving scoring coefficients for the summary scores, as when the negative standardized subscale scores have to be multiplied by negative coefficients, the result will be positive. This effect could generate inconsistencies between the responses at the sub-scale level and the summary scores with possible reflections on the interpretation [29]. In our opinion, further research and consensus studies are required to overcome the possible limitations related to the summary scoring procedure of SF-12.

The evidence regarding the validity and reliability of SF-12 in patients with MFS is important, considering that it could be a valid alternative to the SF-36, which could be too long to administer to patients in clinical practice or studies with large samples [30, 31]. The recent literature also presented version 2 of the SF-12 [32]. The updated version presented several changes from version one, including the wording of some items, and the response options of discrete items were reduced from six- to five-response options. The updated version of SF-12 does not exclude the use in clinical practice and research of the first version of the scale. Although the validation of version 2 of SF-12 could interesting for the research field, we decided to validate version one of the SF-12 due to it is still the most used vision in the clinical practice [26] and, accordingly, clinicians require an evidence-grounded base when they adopt the SF-12 in patients with MFS. Future investigations should confirm the structural validity and reliability of version 2 of SF-12 among adults with MFS.

Finally, considering the summary scores derived from this study, we noticed that the mean score of PCS12 (mean = 45.93; SD = 10.79) was lower than the PCS12 recently reported in Italian patients with an equivalent age distribution and affected moderate or complex congenital heart diseases [PCS12_mean score_ = 48.69; SD_PCS12_ = 8.96; MCS12_mean score_ = 45.56; SD_MCS12_ = 10.99) [33]. Conversely, the mean score of MCS12 reported in this study (mean = 45.41; SD = 10.17) was similar to the one reported in adults with moderate or complex congenital heart diseases (mean = 45.56; SD = 10.99) [33]. More precisely, the mean PCS12 score exhibited in this study derived from patients with MFS who had a mean age of 42.91 years (SD = 12.19) was similar to the PCS12 reported in Italian older adults (mean age of 75 years; SD = 10.2) with chronic morbidities [34], who reported having a mean PCS12 score of 44 (SD = 9.6). In the general Italian population, it was determined that for cohorts with a mean age of 44 years, the mean PCS12 score was reported to be equal to 52.7(SD = 6.0), and the mean MCS12 score was equal to 48.2 (SD = 9.8) [15]. Overall, our results are also consistent with previous research on HRQoL among patients with MFS [5, 35] that highlighted the need for greater attention towards the research endeavours aimed at describing the determinants of HRQoL to provide guidance for planning specific supportive educational strategies for enhancing self-resources and, consequently, the HRQoL of patients with MFS.

This study has several limitations. First, the sample size is limited if compared to other SF-12 validation studies [8]; however, we have to acknowledge that the MFS is a rare condition, and the desired sample size of this study was determined for performing a factor analysis considering 50 participants per factor as adequate [10]. Second, the sample size did not allow to perform possible additional sub-group analyses, as per previous research [9], such as testing the model invariance between patients from different age groups, different educational backgrounds, and between females and males. Third, the data were collected using a cross-sectional approach; therefore, we have no information on the patients’ responses over time. Fourth, the investigation was carried out in Italy; thus, we require cross-national research for providing more generalizable evidence regarding the validity and reliability of the SF-12 in the population affected by MFS.

## Conclusions

This study confirmed the structural validity and the reliability of SF-12 among adults with MFS. Therefore, SF-12 could be a suitable solution for assessing HRQoL in patients with MFS, especially when it is required a self-report tool that needs to be compiled in a limited amount of time for both research purposes and clinical practice. Future cross-national and multi-center research should confirm the SF12 measurement invariance among patients with MFS in different national contexts and considering the test of the invariance in relation to specific socio-demographic characteristics, such as educational level, age groups, and sex.

## Supporting information

S1 Table Associations between socio-demographic characteristics and PCS12/MCS12. (DOCX)
