# Supplementary material for: Is SF-12 a valid and reliable measurement of health-related quality of life among adults with Marfan syndrome? A confirmatory study
Source: PLoS One. 2021 Jun 9;16(6):e0252864. doi: 10.1371/journal.pone.0252864 (PMC8189474; doi:10.1371/journal.pone.0252864)
Supplement: S1 Table — (DOCX) [file pone.0252864.s001.docx]

| **S1 Table**  *Associations between socio-demographic characteristics and PCS12/MCS12* | | | | | | | | | | |
| --- | --- | --- | --- | --- | --- | --- | --- | --- | --- | --- |
|  |  | PCS12 | | | | MCS12 | | | | |
|  |  | β | 95%CI | | *p* | β | 95%CI | | *p* |  |
| *Independent variables* | |  |  |  |  |  |  |  |  |  |
|  | Sex (1=male; 2=female) | -.092 | -6.047 | 2.041 | 0.328 | -.072 | -5.529 | 2.545 | .465 |  |
|  | Age | -.324 | -.464 | -.109 | 0.002 | .091 | -.101 | .253 | .395 |  |
|  | Education (lower versus higher) | .049 | .599 | -1.929 | 3.325 | .058 | -1.845 | 3.399 | .558 |  |
|  | Years from diagnosis | .082 | -.085 | .210 | .403 | -.074 | -.201 | .094 | .476 |  |
| *Model* | |  |  |  |  |  |  |  |  |  |
|  | Test F (*p*) | 3.659(0.008) | | | | 0.373 (0.827) | | | | |
| R^2^ | | 0.348 | | | | 0.014 | | | | |
